# Exploration of the Healthy Donor Effect Among 0.6 Million Blood Donors in China: Longitudinal Study

**DOI:** 10.2196/48617

**Published:** 2024-02-22

**Authors:** Shu Su, Yang Sun, Xiaoyun Gu, Wenjie Wu, Xiaodong Su, Ting Ma, Aowei Song, Xinxin Xie, Liqin Wang, Qianke Cheng, Lingxia Guo, Lei Zhang, Jiangcun Yang

**Affiliations:** 1 Department of Transfusion Medicine Shaanxi Provincial People’s Hospital Xi'an China; 2 Department of Epidemiology and Biostatistics The Second Affiliated Hospital of Chongqing Medical University Chong Qing China; 3 Data Center Shaanxi Provincial People’s Hospital Xi'an China; 4 Department of Information Technology Shaanxi Health Information Center Xi'an China; 5 Department of Medical Record Management Shaanxi Provincial People’s Hospital Xi'an China; 6 Planning Development and Information Office Health Commission of Shaanxi Province Xi'an China; 7 China-Australia Joint Research Center for Infectious Diseases School of Public Health Xi’an Jiaotong University Health Science Center Xi'an China; 8 Artificial Intelligence and Modelling in Epidemiology Program Melbourne Sexual Health Centre Alfred Health Melbourne Australia; 9 Central Clinical School Faculty of Medicine Monash University Melbourne Australia

**Keywords:** healthy donor effect, blood donors, predonation, health profile, longitudinal study, propensity score matching

## Abstract

**Background:**

The World Health Organization emphasizes the importance of completely voluntary blood donation to maintain safe and sustainable blood supplies. However, the benefits of blood donation for donors, such as reducing the risk of disease, remain a topic of debate due to the existence of the healthy donor effect (HDE). This effect arises because of inherent health differences between blood donors and the general population, and it is also considered a methodological issue.

**Objective:**

This study aims to generate a more detailed health profile of blood donors from a donor cohort study to mitigate and quantify the HDE and properly interpret the association between blood donation and disease outcomes among blood donors.

**Methods:**

A retrospective cohort study was conducted between January 2012 and December 2018 among donors before their first donation. One-to-one propensity score matching was conducted through a random selection of individuals without any history of blood donation, as reported from their electronic health records. We conducted a Poisson regression between blood donors and non–blood donors before the first donation to estimate the adjusted incidence rate ratio (AIRR) of selected blood donation–related diseases, as defined by 13 categories of *International Classification of Diseases, Tenth Revision* (ICD-10) codes.

**Results:**

Of the 0.6 million blood donors, 15,115 had an inpatient record before their first donation, whereas 17,356 non–blood donors had an inpatient record. For the comparison between blood donors and the matched non–blood donors, the HDE (the disease incidence rate ratio between non–blood donors and blood donors) was an AIRR of 1.152 (95% CI 1.127-1.178; *P*<.001). Among disease categories not recommended for blood donation in China, the strongest HDE was observed in the ICD-10 D50-D89 codes, which pertain to diseases of the blood and blood-forming organs as well as certain disorders involving the immune mechanism (AIRR 3.225, 95% CI 2.402-4.330; *P*<.001). After age stratification, we found that people who had their first blood donation between 46-55 years old had the strongest HDE (AIRR 1.816, 95% CI 1.707-1.932; *P*<.001). Both male and female donors had significant HDE (AIRR 1.082, 95% CI 1.05-1.116; *P*=.003; and AIRR 1.236, 95% CI 1.196-1.277; *P*<.001, respectively) compared with matched non–blood donors.

**Conclusions:**

**:** Our research findings suggest that the HDE is present among blood donors, particularly among female donors and those who first donated blood between the ages of 46 and 55 years.

**Trial Registration:**

Chinese Clinical Trial Registry ChiCTR2200055983; https://www.chictr.org.cn/showproj.html?proj=51760

## Introduction

The World Health Organization (WHO) promotes voluntary blood donation as the safest and most effective means to maintain an adequate supply of safe blood and blood products for transfusion. To ensure the safety and sustainability of blood supplies, the WHO advocates for completely voluntary blood donation without compensation. Despite the collection of approximately 118.54 million blood donations worldwide [1], the benefits of blood donation for donors are still a matter of debate, as some studies suggest the existence of the healthy donor effect (HDE).

The HDE refers to the observation that blood donors exhibit lower disease morbidity and mortality compared with the general population. This phenomenon can be attributed to the fact that blood donors are typically selected from a healthier subset of the population, due to both donor selection procedures and self-selection factors [2,3]. The HDE has been examined in numerous past studies by comparing the health status of blood donors to that of non–blood donors [3-8]. Although some studies have failed to reveal any significant effects on health status resulting from blood donation [4,6,7], others found substantial differences such as decreased cancer incidence [5] and better cardiovascular status in blood donors compared with the general population [9]. The findings regarding the HDE have been largely inconclusive and sometimes contradictory, mainly because researchers were not able to deal with inevitable selection bias, that is, the fact that donors have better healthier status due to the donation policy requirements. In practice, it is nearly impossible to determine whether a beneficial health status observed in blood donors reflects the inherent healthier status of donors or the actual health gains due to blood donation [8]. Thus, it is important to mitigate and quantify the HDE to properly interpret the association between blood donation and disease outcomes.

Multiple methods have been used to address the HDE in various studies. The main strategy is to apply an exposure window or qualification period to potentially mitigate the HDE [10]. It mainly involves using 1 qualification period (ie, all donors must donate for at least 5 or 10 years to be eligible for the study) to adjust for the HDE [11-13]. However, this effort to reduce the HDE is conducted after blood donation. In addition, some studies reduce selection bias by analyzing only healthier groups; comparing differences within blood donors; and dealing with mixed bias, including the HDE, through ANOVA or some form of regression analysis [14-17]. However, adjusting age, sex, and demographic factors to reduce the HDE through some form of statistical strategy will affect the probability of subsequent exposure, and adjusting for confounding factors is not enough to address the HDE sufficiently [15,16]. To avoid the possible bias, another method is to compare or restrict the study within the active donor population, which still cannot exclude the existence of the residual HDE from most observational studies [14,17,18]. Currently, there is no evidence to certify the existence of the HDE prior to donation. The major weakness is that the potential HDE is not quantified and is usually estimated by researchers [10].

The first step to reduce the HDE is to accurately estimate it. The precise assessment of the HDE can be retrospectively obtained during the predonation period. Leveraging our large blood donor cohort, which is among the largest in the world, we are able to match non–blood donors and thus overcome the major limitations of previous studies on the HDE. This would allow us to obtain unprecedented insights into the health status of blood donors and non–blood donors and to quantify the HDE in a more accurate manner. Our study was conducted as a retrospective cohort study, wherein the health status of blood donors and non–blood donors were compared by one-to-one propensity score matching (PSM). Our main objective is to confirm the existence of the HDE in the population while also assessing the impacts of age and sex on the HDE. Through this comprehensive analysis, we aim to generate a more detailed health profile of blood donors to certify the HDE and better understand the underlying factors that contribute to it.

## Methods

### Data Source

We extracted the disease information from blood donors in Shaanxi Province, which is located in Northwest China. The Shaanxi Blood Donation Database is a computerized, combined donation and transfusion register from 3.4 million individuals who donated blood voluntarily between 1998-2018, and their relevant blood information was collected in the Shaanxi Blood Donor Database by linking with province-wide Shaanxi electronic health records (EHRs) and centralized hospital medical records. From this linkage, all the inpatient records of the donors can be searched. This database also tracks the disease information of participants without any history of blood donation. We have provided detail information for this cohort in our previous publication [19]. The disease burden of blood donors and non–blood donors was demonstrated. This natural and representative population cohort could be used to describe the health profiles of blood donors and investigate the potential HDE for selected clinically diagnosed diseases (as listed below) for donors, compared with non–blood donors, before the first donation date.

**Figure 1:**
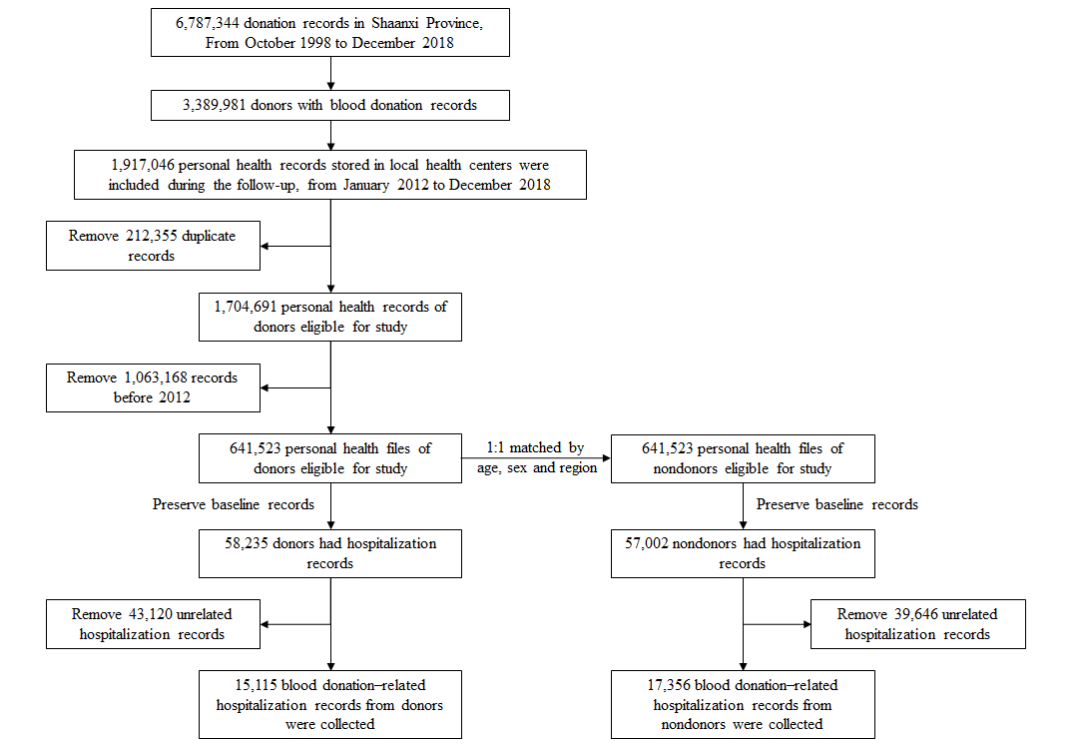
Study flowchart of the longitudinal study.

We initially included 3,389,981 donors in the cohort, and 1,907,146 had health records from January 1, 2012, to December 31, 2018. We excluded 1,063,418 participants with disease records before 2012, resulting in 641,523 qualified EHR records of eligible donors. Based on this information, we established a control group with the same criteria by using one-to-one matching to the donor group. Our study recognized the substantial impact of factors such as population aging, sex disparities, environmental issues, and rural-urban differences on individual health [20-23]. Thus, to provide a more accurate assessment of the HDE, we used a PSM analysis. This approach allowed us to match non–blood donors to blood donors with identical demographic characteristics (sex, age, and residence) from the EHRs and investigate the occurrence of a wide range of inpatient diseases defined by *International Classification of Diseases, Tenth Revision* (ICD-10) codes prior to the first donation date. The number of disease records was calculated during 2012-2018 and was extracted from the inpatient record, which is automatically uploaded from the hospital system. The matching was conducted by random sampling without the replacement of individuals without any history of blood donation from the EHR. We only extracted the disease information related to the blood donation criteria, resulting in the blood donor data set and non–blood donor data set having 58,235 and 57,002 inpatient records, respectively. Then, we further restricted the disease records to before the donation period, resulting in 15,115 donor records and 17,356 nondonor records, respectively (Figure 1).

### Selection of Diseases

According to the national guidelines for blood donation requirements [24], only specific diseases that are related to blood donation were selected for study. Thus, diseases corresponding to injury, poisoning, pregnancy, childbirth and the puerperium, and others were excluded. We only collected disease information for 13 categories—certain infectious and parasitic diseases (A00-B99); neoplasms (C00-D48); diseases of the blood and blood-forming organs and certain disorders involving the immune mechanism (D50-D89); endocrine, nutritional, and metabolic diseases (E00-E90); mental and behavioral disorders (F00-F99); diseases of the nervous system (G00-G99); diseases of the circulatory system (I00-I99); diseases of the respiratory system (J00-J99); diseases of the digestive system (K00-K93); diseases of the skin and subcutaneous tissue (L00-L99); diseases of the genitourinary system (N00-N99); external causes of morbidity and mortality (V01-Y98); and factors influencing health status and contact with health services (Z00-Z99)—to conduct the comparison between blood donors and non–blood donors before donation.

### Inclusion and Exclusion Criteria

In this cohort, we only included (1) blood donors who gave their first donation between 2012 and 2018 and (2) blood donors who have EHR records between 2012 and 2018 in Shaanxi Province. We excluded (1) blood donors without a blood donation date; (2) blood donors with a disease record before 2012 or who left Shaanxi Province; and (3) blood donors with incomplete demographic information. For the control group of non–blood donors, identical inclusion and exclusion criteria were implemented, with the exception of content specific to blood donation. This standardized approach ensures comparability between the blood donor and non–blood donor cohorts, establishing a robust foundation for our comprehensive investigation into the HDE.

### Assessing the HDE

**Figure 2:**
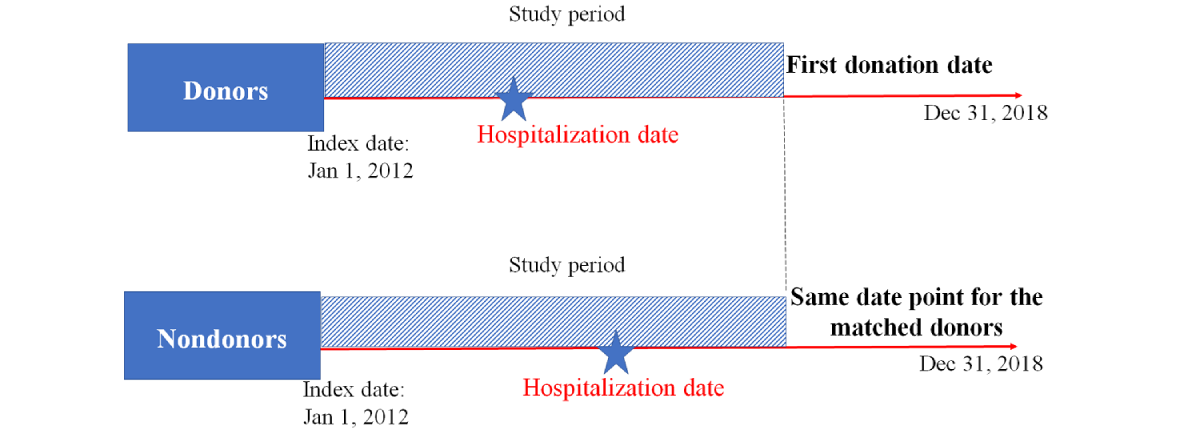
Study schematic of the longitudinal study.

We then specifically collected the disease information of these blood donors between baseline and the date of their first blood donation. Similarly, we also extracted the disease information of the matching non–blood donors during the same time and compared the disease rates to quantify the HDE. For example, we collected the number of certain infectious and parasitic diseases (A00-B99) diagnosed among donors and nondonors during the study period, which was used as the numerator, whereas the total observed number of donors and nondonors (it is the same number in this study; n=641,523) was used as the denominator (Figure 2). Then, we further estimated the incidence rate ratio between these 2 groups.

According to the Chinese blood donation guidelines, we set the age criteria for entering the cohort as 18-55 years, and we stratified age into 4 groups (18-25, 26-35, 36-45, and 46-55 years). The age when the participants entered the cohort was used. The model compared all the disease rates among male and female donors and nondonors among the 4 age groups. Poisson regression models were built to identify donors’ and nondonors’ health status in the respective exposure windows. The observed period was defined as from 2012 to the first donation date, and the non–blood donors used the same cutoff point accordingly (Figure 2).

### Statistical Analysis

Poisson regression is often used for count data where the outcome variable represents the number of events that occur in a fixed interval of time or space. It is also appropriate for rare events [25]. This methodology has been widely used in HDE studies [3,26]. We used Poisson regression to compare the likelihood of becoming hospitalized between blood donors and non–blood donors prior to the first blood donation. The adjusted incidence rate ratio (AIRR) was used as an estimate of the ratio of the incident risks for an event for the non–blood donors to the risks for the blood donors during the study period; that is, the reference group is blood donors. The model was adjusted by occupation, education, and marital status. The waiting period between hospital discharge and first blood donation was calculated for each age group in each disease category. Descriptive and inferential statistical analyses were conducted as part of our methodology. Mean and SD were used to summarize age and donation counts, whereas median and IQR were used to summarize the duration in days between discharge and the first donation. For categorical variables, we described them using frequencies and percentages. In the context of inferential statistical tests, we used both multivariate regression analyses and sensitive analysis to evaluate model assumptions and validate the suitability of our chosen analytical techniques. All statistical analyses and data handling were conducted with computer software (SAS version 9.4; SAS Institute).

### Ethical Considerations

Ethical approval for the study was obtained from the institutional review board of the People’s Hospital of Shaanxi Province (No: 2020-R002). The study was preregistered at China National Medical Research Register (MR-61-21-011750) and Chinese Clinical Trial Registry (ChiCTR2200055983). Since our study is a secondary analysis using existing data with primary consent, where the original consent or institutional review board approval covers secondary analysis, no additional consent was needed for this study. The data have been deidentified and can only be accessed by the researcher group for this study.

## Results

### Baseline Characteristics

This study included 641,523 blood donors and 641,523 non–blood donors, which were matched by age, sex, and region. There were 396,681 male donors and 244,842 female donors in the study, and a total of 3,396,992 person-years (PYs) were observed. The mean age was 30.4 (SD 9.3) years, with most of them (253,045/641,523, 39.4% for both groups) being aged 18-25 years. The majority (429,421/641,523, 66.9%) of the blood donors came from Central Shaanxi, 26.6% (170,274/641,523) came from South Shaanxi, and 6.5% (41,828/641,523) came from North Shaanxi. Age, sex, and region distributions of the non–blood donors were the same as those of blood donors. Regarding education, 59.2% (379,592/641,523) of the blood donors and 56.9% (365,294/641,523) of non–blood donors had received an education of junior high school or below. Regarding occupation, 65.3% (418,757/641,523) of the blood donors and 61.2% (392,540/641,523) of the non–blood donors were farmers. Regarding marriage status, 71.4% (458,070/641,523) of the blood donors and 70.5% (452,156/641,523) of the non–blood donors were married. Baseline characteristics of the participants are displayed in Table 1.

**Table 1:** Basic demographic characteristics of participants stratified by blood donors and non-blood donors.

Characteristics		Blood donors (n=641,523)	Non–blood donors (n=641,523)	*P* value^a^	
**Sex, n (%)**					>.99
	Male	396,681 (61.8)	396,681 (61.8)		
	Female	244,842 (38.2)	244,842 (38.2)		
**Age (years), mean (SD)**		30.4 (9.3)	30.4 (9.3)	>.99	
	18-25, n (%)	253,045 (39.4)	253,045 (39.4)		
	26-35, n (%)	180,185 (28.1)	180,185 (28.1)		
	36-45, n (%)	164,645 (25.7)	164,645 (25.7)		
	46-55, n (%)	43,648 (6.8)	43,648 (6.8)		
Number of donation, mean (SD)		1.45 (1.07)	0 (0)		
**Education, n (%)**					<.001
	Junior high and below	379,592 (59.2)	365,294 (56.9)		
	Senior high	119,669 (18.7)	114,611 (17.9)		
	University and above	64,081 (10)	81,930 (12.8)		
	Unknown	78,181 (12.2)	79,688 (12.4)		
**Marriage status, n (%)**					<.001
	Single	132,613 (20.7)	139,177 (21.7)		
	Married	458,070 (71.4)	452,156 (70.5)		
	Divorced	8007 (1.2)	7729 (1.2)		
	Unknown	42,833 (6.7)	42,461 (6.6)		
**Occupation, n (%)**					<.001
	Worker	74,266 (11.6)	73,061 (11.4)		
	Farmer	418,757 (65.3)	392,540 (61.2)		
	Self-employed	121,609 (19)	140,808 (21.9)		
	Unknown	26,891 (4.2)	35,114 (5.5)		
**Region, n (%)**					>.99
	North Shaanxi	41,828 (6.5)	41,828 (6.5)		
	Central Shaanxi	429,421 (66.9)	429,421 (66.9)		
	South Shaanxi	170,274 (26.5)	170,274 (26.5)		

^a^Chi-square test.

### Disease Burden Among Donors

Among the blood donors, 15,115 participants had inpatient records during the observed period, with a median follow-up duration of 1061 (IQR 510-1803) days. The disease incidence consistently showed an upward trend with age: it increased from 26.69 per 10,000 PYs for the 18-25 years age group to 86.98 per 10,000 PYs for the 46-55 years age group. Diseases of the digestive system (K00-K93) were most reported diseases among blood donors with 3240 records, which was followed by diseases of the circulatory system (I00-I99; n=2856) and diseases of the genitourinary system (N00-N99; n=2519). Diseases of the circulatory system (I00-I99) were the most reported diseases with an incidence rate of 30.30 per 10,000 PYs in the 46-55 years age group.

### Disease Burden Among Non–Blood Donors

Among non–blood donors, 17,356 participants had inpatient records during the observed period. The disease incidence increased with age: it ranged from 34.63 per 10,000 PYs for the 18-25 years age group to 149.51 per 10,000 PYs for the 46-55 years age group. There were 3462 patients who had diseases of the digestive system (K00-K93), which was the most reported disease, followed by diseases of the circulatory system (I00-I99; n=3042) and diseases of the genitourinary system (N00-N99; n=2533). The highest incidence of 44.36 per 10,000 PYs was found among diseases of the circulatory system (I00-I99) in the 46-55 years age group.

### HDE on the Overall Population

**Figure 3:**
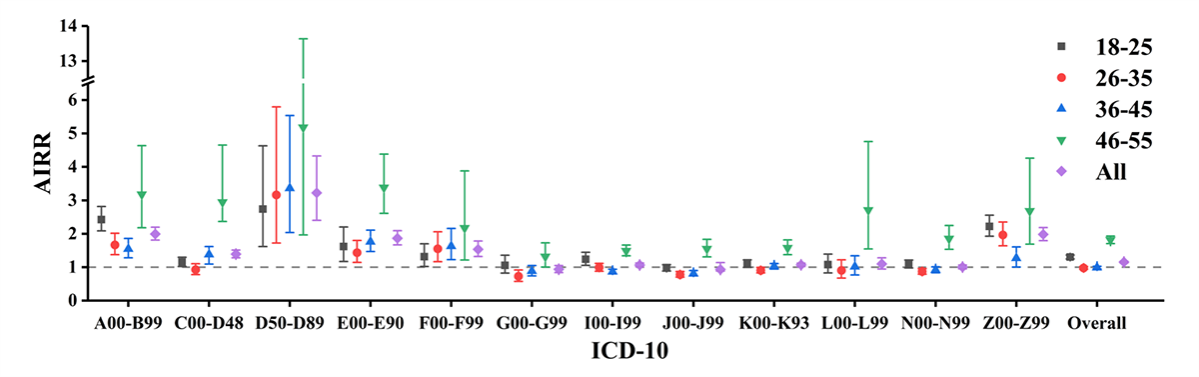
Adjusted incidence rate ratio (AIRR) in comparing blood donation–related diseases between donors and nondonors: the healthy donor effect. ICD-10: *International Classification of Diseases, Tenth Revision*.

Overall, the risk of reporting ICD-10 diseases in nondonors was generally higher than that from donors before the first blood donation (AIRR 1.152, 95% CI 1.127-1.178; *P*<.001), which was defined as the existence of HDE. The top 3 diseases with the strongest HDE were observed in diseases of the blood and blood-forming organs and certain disorders involving the immune mechanism (D50-D89; AIRR 3.225, 95% CI 2.402-4.330; *P*<.001); certain infectious and parasitic diseases (A00-B99; AIRR 1.995, 95% CI 1.812-2.196; *P*<.001); and factors influencing health status and contact with health services (Z00-Z99; AIRR 1.980, 95% CI 1.796-2.184; *P*<.001). When considering the specific diseases, non–blood donors had a lower risk in relation to diseases of the nervous system (G00-G99; AIRR 0.939, 95% CI 0.841-1.049; *P*=.13); diseases of the respiratory system (J00-J99; AIRR 0.933, 95% CI 0.882-1.135; *P*=.29); diseases of the skin and subcutaneous tissue (L00-L99; AIRR 1.101, 95% CI 0.946-1.281; *P*=.21); and diseases of the genitourinary system (N00-N99; AIRR 1.006, 95% CI 0.952-1.064; *P*=.32; Figure 3 and Table S1 in Multimedia Appendix 1).

### HDE by Sex Differences

**Figure 4:**
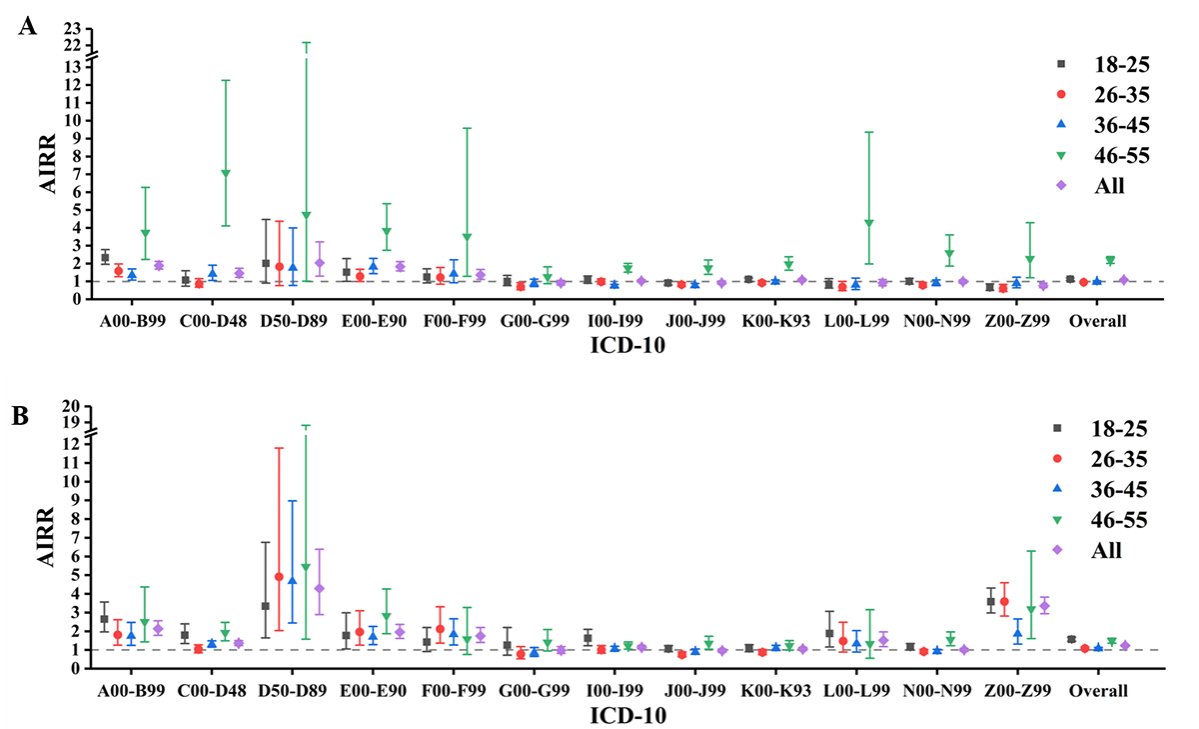
Adjusted incidence rate ratio (AIRR) in comparing blood donation–related diseases between donors and nondonors by sex—(A) male and (B) female: the healthy donor effect. ICD-10: *International Classification of Diseases, Tenth Revision*.

Our study found that male and female individuals both have significant HDE, but it was stronger in female individuals (AIRR 1.236, 95% CI 1.196-1.277; *P*<.001) than male individuals (AIRR 1.082, 95% CI 1.050-1.116; *P*=.003). The most substantial difference was found in the risk of having factors influencing health status and contact with health services (Z00-Z99), as female non–blood donors in all age groups had a higher risk than blood donors, but only male non–blood donors aged 46-55 years had a higher risk (AIRR 2.273, 95% CI 1.205-4.289; *P*<.001). For diseases, the most significant difference was observed in the risk of diseases of the blood and blood-forming organs and certain disorders involving the immune mechanism (D50-D89): the HDE of female donors was stronger than that of male donors (female: AIRR 4.294, 95% CI 2.887-6.388; *P*<.001 vs male: AIRR 2.042, 95% CI 1.299-3.210; *P*<.001). After age stratification, male donors who had their first donation between 46-55 years old had the strongest HDE among all age groups (AIRR 2.193, 95% CI 2.011-2.392; *P*<.001), whereas female donors who first donated between 18-25 years old had the strongest HDE (AIRR 1.571, 95% CI 1.465-1.685; *P*<.001; Figure 4 and Tables S2-3 in Multimedia Appendix 1).

### HDE by Age Groups

In terms of age groups, among non–blood donors, the 46-55 years age group showed the highest risk (AIRR 1.816, 95% CI 1.707-1.932; *P*<.001), followed by the 18-25 years age group (AIRR 1.302, 95% CI 1.245-1.362; *P*<.001). Conversely, the 26-35 and 36-45 years age groups did not show significant differences between donors and nondonors (AIRR 0.979, 95% CI 0.936-1.023; *P*=.21 and AIRR 0.988, 95% CI 0.951-1.025; *P*=.32, respectively). Notably, donors who initiated donation after the age of 46 years had a consistently better health status compared with non–blood donors. It was shown that non–blood donors had a 5-times higher risk than donors in diseases of the blood and blood-forming organs and certain disorders involving the immune mechanism (D50-D89; AIRR 5.181, 95% CI 1.968-13.636; *P*<.001) and a 3-times higher risk in endocrine, nutritional, and metabolic diseases (E00-E90; AIRR 3.384, 95% CI 2.612-4.383; *P*<.001) and certain infectious and parasitic diseases (A00-B99; AIRR 3.181, 95% CI 2.183-4.635; *P*<.001). Non–blood donors in all age classes were associated with higher risks for certain infectious and parasitic diseases (A00-B99); diseases of the blood and blood-forming organs and certain disorders involving the immune mechanism (D50-D89); endocrine, nutritional, and metabolic diseases (E00-E90); mental and behavioral disorders (F00-F99); and factors influencing health status and contact with health services (Z00-Z99) compared with donors (Figure 3 and Table S1 in Multimedia Appendix 1).

In contrast, non–blood donors aged 26-35 years had a lower risk of diseases of the nervous system (G00-G99; AIRR 0.728, 95% CI 0.576-0.919; *P*<.001); diseases of the respiratory system (J00-J99; AIRR 0.781, 95% CI 0.699-0.87; *P*<.001); and diseases of the digestive system (K00-K93; AIRR 0.907, 95% CI 0.827-0.995; *P*=.007). Non–blood donors aged 36-45 years only had a lower risk for diseases of the circulatory system (I00-I99; AIRR 0.866, 95% CI 0.802-0.936; *P*<.001) and diseases of the respiratory system (J00-J99; AIRR 0.805, 95% CI 0.738-0.901; *P*<.001; Figure 3 and Table S1 in Multimedia Appendix 1).

### Sensitive Analysis

Upon conducting a comparative analysis, we made necessary adjustments to the overall findings by excluding the disease categories of external causes of morbidity and mortality (V01-Y98) and factors influencing health status and contact with health services (Z00-Z99). Subsequently, our results demonstrated a consistent increase in the risk of HDE among both blood donors and non–blood donors, with an AIRR of 1.117 (95% CI 1.092-1.143).

### Gaps Between Hospital Discharge and First Blood Donation

The overall median waiting period between inpatients being discharged from the hospital and the first blood donation was 534 (IQR 241-960) days. This duration showed a declining trend with respect to age groups. The longest waiting period was found in the 18-25 years age group (median 592, IQR 267-1027 days). The shortest waiting period was found in the 46-55 years age group (median 439, IQR 204-813 days). In the 26-35 years age group, the gap was a median of 531 (IQR 240-970) days, whereas in the 36-45 years age group, the gap was a median of 521 (IQR 239-942) days. The longest gap was observed among those who had the disease category of neoplasms (C00-D48; median 673, IQR 358-1137 days), followed by factors influencing health status and contact with health services (Z00-Z99; median 660, IQR 373-1109 days), and certain infectious and parasitic diseases (A00-B99; median 654, IQR 298-1154 days). The shortest gap was found in those with the disease category of endocrine, nutritional, and metabolic diseases (E00-E90; median 456, IQR 152-877 days). Regarding the overall trend, the waiting period showed a descending trend, with the gap changing from a median of 592 (IQR 267-1027) days in 18-25 years age group to a median of 439 (IQR 204-813) days in the 46-55 years age group (Table 2).

**Table 2:** Gaps between hospital discharge and the first blood donation among blood donors.

Disease categories	Days between discharge and the first donation, median (IQR)				
	18-25 years age group	26-35 years age group	36-45 years age group	46-55 years age group	All age groups
Certain infectious and parasitic diseases (A00-B99)	770 (335-1192)	700 (347-1134)	552 (278-1074)	415 (259-872)	654 (298-1154)
Neoplasms (C00-D48)	595 (286-978)	583 (301-991)	726 (405-1173)	698 (329-1103)	673 (358-1137)
Diseases of the blood and blood-forming organs and certain disorders involving the immune mechanism (D50-D89)	496 (262-916)	592 (272-1292)	524 (277-1022)	485 (272-988)	510 (272-1069)
Endocrine, nutritional, and metabolic diseases (E00-E90)	451 (216-828)	496 (142-926)	493 (182-893)	273 (75-653)	456 (152-877)
Mental and behavioral disorders (F00-F99)	533 (301-940)	567 (267-1063)	598 (291-1140)	367 (87-1007)	560 (276-998)
Diseases of the nervous system (G00-G99)	472 (164-953)	544 (256-992)	471 (220-888)	406 (159-787)	473 (205-896)
Diseases of the circulatory system (I00-I99)	608 (256-1054)	477 (208-936)	497 (204-921)	413 (200-796)	481 (206-910)
Diseases of the respiratory system (J00-J99)	649 (291-1124)	571 (234-1002)	571 (273-979)	429 (230-938)	582 (260-1037)
Diseases of the digestive system (K00-K93)	604 (263-1023)	583 (267-1034)	610 (310-1031)	537 (303-855)	593 (285-1011)
Diseases of the skin and subcutaneous tissue (L00-L99)	432 (178-999)	573 (267-1052)	545 (239-934)	638 (226-916)	523 (232-969)
Diseases of the genitourinary system (N00-N99)	611 (259-1052)	551 (241-1007)	605 (275-1070)	581 (242-961)	584 (260-1036)
External causes of morbidity and mortality (V01-Y98)	N/A^a^	N/A	N/A	N/A	N/A
Factors influencing health status and contact with health services (Z00-Z99)	661 (378-1054)	730 (409-1216)	632 (313-1081)	580 (344-956)	660 (373-1109)
Overall	592 (267-1027)	531 (240-970)	521 (239-942)	439 (204-813)	534 (241-960)

^a^N/A: not applicable.

## Discussion

The findings of our study provide robust evidence of the existence of the HDE among blood donors in China. In line with the blood donation guidelines of the country, the overall health status of blood donors was found to be superior to that of non–blood donors, as evidenced by a reduced risk of developing selected diseases. In particular, individuals who had their first blood donation at or after the age of 46 years exhibited a significantly lower risk of developing diseases across all categories compared with non–blood donors. The results of our study highlight a marked disparity in the HDE by sex, with concerns that donating blood may negatively impact the health of participants with these conditions. Although previous research has aimed to disprove the HDE, our study is the first to both confirm its presence and quantify its magnitude.

Although blood donors generally exhibit a better health status than non–blood donors during the predonation period, our findings indicate that non–blood donors are superior in certain disease age groups. The disparity in health status between blood donors and non–blood donors could be attributed to several factors. First, our data revealed that blood donors with a higher disease rate than non–blood donors were all younger than the age of 45 years. This observation suggests that older donors may be more conscientious of their health and well-being [27,28]. Second, certain diseases, although they may require hospitalization, do not necessarily prohibit patients from donating blood. For instance, our data revealed that the majority of blood donors with inpatient records for respiratory diseases had contracted influenza or pneumonia (J09-J18). As the risk of transmitting these diseases directly through blood or blood products is extremely low, individuals who have recovered from these conditions and are free from clinical symptoms may still be eligible to donate blood [29]. Third, previous studies have shown that appropriate blood donation can help reduce the risk of certain diseases. Blood donation has been found to increase the concentrations of high-density lipoprotein and apolipoprotein A while also lowering the potential for oxidation of low-density lipoprotein (LDL) particles. Lower levels of LDL peroxidation result in higher oxidation potential and increased concentration of nitric oxide in LDL particles, which may have a protective effect against certain diseases [30]. Additionally, blood donation has been associated with a reduced risk of circulatory diseases and tumors [31,32], possibly due to the reduction of iron concentrations in the body through blood loss therapy. However, some studies suggest that iron loss through blood donation may also be associated with certain diseases [6,33,34].

This study demonstrated that female individuals exhibited a stronger HDE than male individuals. This difference can be attributed to social and physiological factors, as well as the different incidence of common diseases in male and female individuals [35]. Furthermore, female individuals demonstrated a strong HDE in relation to oncology and digestive diseases, which may be related to life circumstances, work stress, and lifestyle factors such as smoking and alcohol consumption [36,37]. Interestingly, the results of age stratification indicated that the strength of the HDE differs between age categories. Specifically, female individuals between the ages of 18-45 years exhibited a stronger HDE than their male counterparts, whereas male individuals aged 46-55 years exhibited a stronger HDE compared to female individuals in the same age group. These results may be explained by the overall physiological differences that exist between male and female individuals in their reproductive stages, such as the menstrual cycle and pregnancy, which could lead to female donors paying more attention to their health and neonatal health and following the donation guidelines more closely, resulting in an increased HDE compared to male individuals [38]. Importantly, it is worth noting that the strength of HDE was no longer accentuated in female individuals after the age of 46 years, as they have entered the nonreproductive phase and the aforementioned reproductive factors have disappeared, resulting in a stronger HDE in male individuals compared to female individuals. These results highlight the importance of considering sex effects in the expression of the HDE.

We also found that the average time interval between participants donating blood and being discharged from hospital was almost 2 years. Given the shortage of blood storage, it is recommended that restrictions on donation requirements be discussed, similar to other countries [39], such as allowing inpatients with cured diseases to donate blood after a certain period. This is especially important during the COVID-19 outbreak, where blood supply shortages have been reported [40], and China has updated guidelines stating that patients with COVID-19 can donate blood after 7 days of seroconversion [41]. We are the first to demonstrate that older people have a shorter interval between discharge and blood donation than younger people, which may be due to 2 reasons. First, as the age limit for blood donation is ≤55 years, donors must donate blood before this age limit. Many countries have a higher age limit than China, and considering that life expectancy has increased in China, the age limit for blood donation may also need to be increased. Furthermore, China encourages donors to donate blood by allowing the donor or a family member to receive an equal volume of the donated blood with priority. Older adults may have been more reluctant to donate blood previously but have become more accepting after this promotion policy was enacted. Consistent with this policy, donors aged 46-55 years have increased from 11.7% in 2012 to 19.6% in 2018 in Shaanxi Province, whereas donors aged 18-25 years have decreased from 36.7% in 2012 to 30.3% in 2018 [42]. However, it is crucial to ensure the safety of blood transfusions, particularly for donors with hospitalization records. To address this, we recommend that blood centers link donors’ identification with their EHRs, allowing health care staff to access their previous disease history and determine their eligibility for blood donation. In China, the National Health Commission has been promoting the establishment of EHRs with various policies and financial support since 2010 [43]. The health system can be effectively used to inform donors about their medical history, and only healthy individuals with eligible medical histories could be qualified to donate blood or blood components.

Some limitations should be noted in this study. First, as a retrospective study, the records were extracted from the EHR, and the number of people who left the province was not recorded, which may have influenced the hospitalization rate. However, based on the current data, the proportion of immigrants from Shaanxi Province is approximately 10%, and this is unlikely to significantly affect the results. Second, environmental factors and family medical history, both of which may have an impact on respiratory diseases and tumors, were not taken into account in the study. Third, blood routine and liver function data were not readily available for non–blood donors, so we cannot include these factors in the PSM process at baseline. Given that blood routine and liver function tests involve multiple indicators, participants with abnormal data in these tests may potentially have asymptomatic diseases that have not yet been diagnosed. This limitation introduces a potential source of confounding bias in our investigation of related diseases. Finally, the duration of hospitalization may have been influenced by the economic and health care quality factors of the admitting hospitals, which could not be considered in the analysis. Despite these limitations, this study is the first and largest population-based study to provide comprehensive evidence of the existence of the HDE, especially for the female donors and donors who donate over the age of 46 years. We highlighted the substantial differences in blood-related diseases between blood donors and non–blood donors prior to donation. Consequently, this has the potential to introduce bias in comparing donor effects between the 2 groups. Future studies should aim to develop more accurate methods to calculate donor effects and eliminate this bias.

## Appendix

Multimedia Appendix 1 Healthy donor effect in the general population and by gender differences.

## Abbreviations

AIRR: adjusted incidence rate ratio
EHR: electronic health record
HDE: healthy donor effect
ICD-10: *International Classification of Diseases, Tenth Revision*
LDL: low-density lipoprotein
PSM: propensity score matching
PY: person-year
WHO: World Health Organization

## Data Availability

The data sets used in this study are not publicly available because they contain information that could compromise research participant privacy, but they are available from the corresponding author upon reasonable request.

## References

[1] (2023). Blood safety and availability. World Health Organisation.

[2] Atsma F, Veldhuizen I, Verbeek A, de Kort W, de Vegt F (2011). Healthy donor effect: its magnitude in health research among blood donors. Transfusion.

[3] van den Hurk K, Zalpuri S, Prinsze FJ, Merz E, de Kort WLAM (2017). Associations of health status with subsequent blood donor behavior-an alternative perspective on the healthy donor effect from Donor InSight. PLoS One.

[4] Muñoz-Bravo C, Gutiérrez-Bedmar M, Gómez-Aracena J, García-Rodríguez A, Navajas J (2013). Iron: protector or risk factor for cardiovascular disease? still controversial. Nutrients.

[5] Merk K, Mattsson B, Mattsson A, Holm G, Gullbring B, Björkholm M (1990). The incidence of cancer among blood donors. Int J Epidemiol.

[6] Edgren G, Reilly M, Hjalgrim H, Tran TN, Rostgaard K, Adami J, Titlestad K, Shanwell A, Melbye M, Nyrén O (2008). Donation frequency, iron loss, and risk of cancer among blood donors. J Natl Cancer Inst.

[7] Zhang X, Ma J, Wu K, Chan AT, Fuchs CS, Giovannucci EL (2012). Blood donation and colorectal cancer incidence and mortality in men. PLoS One.

[8] Atsma F, de Vegt F (2011). The healthy donor effect: a matter of selection bias and confounding. Transfusion.

[9] Atsma F, Veldhuizen I, de Vegt F, Doggen C, de Kort W (2011). Cardiovascular and demographic characteristics in whole blood and plasma donors: results from the Donor InSight study. Transfusion.

[10] Rahman MM, Karki S, Hayen A (2022). A methods review of the "healthy donor effect" in studies of long-term health outcomes in blood donors. Transfusion.

[11] Peffer K (2015). Blood donation and cardiovascular disease. addressing the healthy donor effect [Dissertation]. Radboud University Nijmegen.

[12] Ullum H, Rostgaard K, Kamper-Jørgensen Mads, Reilly M, Melbye M, Nyrén Olof, Norda R, Edgren G, Hjalgrim H (2015). Blood donation and blood donor mortality after adjustment for a healthy donor effect. Transfusion.

[13] Zhao J, Gabriel E, Norda R, Höglund P, Baden L, Diedrich BA, Marits P, Enoksson SL, Gansner JM, Kaufman R, Dickman PW, Edgren G (2021). Frequent platelet donation is associated with lymphopenia and risk of infections: a nationwide cohort study. Transfusion.

[14] Zhao J, Dahlén Torsten, Brynolf A, Edgren G (2020). Risk of hematological malignancy in blood donors: a nationwide cohort study. Transfusion.

[15] Edgren G, Nyrén Olof, Hultcrantz M, Nielsen KR, Pedersen OB, Björkholm Magnus, Rostgaard K, Hjalgrim H (2016). Blood donation and risk of polycythemia vera. Transfusion.

[16] Amrein K, Katschnig C, Sipurzynski S, Stojakovic T, Lanzer G, Stach E, Pieber TR, Dobnig H (2010). Apheresis affects bone and mineral metabolism. Bone.

[17] Boot CL, Luken JS, van den Burg PJM, de Kort WLAM, Koopman MMW, Vrielink H, van Schoor NM, den Heijer M, Lips P (2015). Bone density in apheresis donors and whole blood donors. Vox Sang.

[18] Shehu E, Hofmann A, Clement M, Langmaack A (2015). Healthy donor effect and satisfaction with health: the role of selection effects related to blood donation behavior. Eur J Health Econ.

[19] Zhang L, Li H, Su S, Wood EM, Ma T, Sun Y, Guo L, Cheng Q, Gu X, Wu W, Wang L, Ding M, Zhang L, Shen Y, Yang J (2022). Cohort Profile: the Shaanxi Blood Donor Cohort in China. Front Cardiovasc Med.

[20] The Lancet (2015). Rural health inequities: data and decisions. Lancet.

[21] Mitchell R, Popham F (2008). Effect of exposure to natural environment on health inequalities: an observational population study. Lancet.

[22] Guo J, Huang X, Dou L, Yan M, Shen T, Tang W, Li J (2022). Aging and aging-related diseases: from molecular mechanisms to interventions and treatments. Signal Transduct Target Ther.

[23] Vlassoff C (2007). Gender differences in determinants and consequences of health and illness. J Health Popul Nutr.

[24] Chinese Ministry of Health (2011). Whole blood and component donor selection requirements. Chinese Standard.

[25] Derby N (2018). An introduction to the analysis of rare events. https://www.pharmasug.org/proceedings/2018/AA/PharmaSUG-2018-AA18.pdf.

[26] Brodersen T, Rostgaard K, Lau CJ, Juel K, Erikstrup C, Nielsen KR, Ostrowski SR, Titlestad K, Saekmose SG, Pedersen OBV, Hjalgrim H (2023). The healthy donor effect and survey participation, becoming a donor and donor career. Transfusion.

[27] Steptoe A, Deaton A, Stone AA (2015). Subjective wellbeing, health, and ageing. Lancet.

[28] Bowling A (2011). Do older and younger people differ in their reported well-being? a national survey of adults in Britain. Fam Pract.

[29] (2006). Maintaining a safe and adequate blood supply in the event of pandemic influenza: guidelines for national blood transfusion services. World Health Organization.

[30] van Jaarsveld H, Pool GF (2002). Beneficial effects of blood donation on high density lipoprotein concentration and the oxidative potential of low density lipoprotein. Atherosclerosis.

[31] Kamhieh-Milz S, Kamhieh-Milz J, Tauchmann Y, Ostermann T, Shah Y, Kalus U, Salama A, Michalsen A (2016). Regular blood donation may help in the management of hypertension: an observational study on 292 blood donors. Transfusion.

[32] Su S, Ma T, Sun Y, Guo L, Su X, Wang W, Xie X, Wang L, Xing L, Zhang L, He S, Yang J, Zhang L (2022). Association between blood donation and malignant and benign tumour risk: a population-based study of 3.4 million participants in China. J Oncol.

[33] Barton JC, Preston BL, McDonnell SM, Rothenberg BE (2001). Severity of iron overload in hemochromatosis: effect of volunteer blood donation before diagnosis. Transfusion.

[34] Ascherio A, Rimm EB, Giovannucci E, Willett WC, Stampfer MJ (2001). Blood donations and risk of coronary heart disease in men. Circulation.

[35] Crimmins E, Shim H, Zhang YS, Kim JK (2019). Differences between men and women in mortality and the health dimensions of the morbidity process. Clin Chem.

[36] Sacco P, Bucholz KK, Harrington D (2014). Gender differences in stressful life events, social support, perceived stress, and alcohol use among older adults: results from a national survey. Subst Use Misuse.

[37] Kontis V, Bennett JE, Mathers CD, Li G, Foreman K, Ezzati M (2017). Future life expectancy in 35 industrialised countries: projections with a Bayesian model ensemble. Lancet.

[38] Moreira LR, Blumenberg C, Caicedo Velasquez BE, Ewerling F, Balandrán A, Vidaletti LP, Varela AR, Hellwig F, Ponce de Leon RG, Barros AJ, Silveira MF, Wehrmeister FC (2023). The role of gender inequality and health expenditure on the coverage of demand for family planning satisfied by modern contraceptives: a multilevel analysis of cross-sectional studies in 14 LAC countries. Lancet Reg Health Am.

[39] Editorial (2019). Is it time to rethink UK restrictions on blood donation?. EClinicalMedicine.

[40] Stanworth SJ, New HV, Apelseth TO, Brunskill S, Cardigan R, Doree C, Germain M, Goldman M, Massey E, Prati D, Shehata N, So-Osman C, Thachil J (2020). Effects of the COVID-19 pandemic on supply and use of blood for transfusion. Lancet Haematol.

[41] General Office of the Health Commission Health (2022). Guidelines for the Prevention and Control of Novel Coronavirus Infection in Blood Stations (Second Edition). National Health Commission of China.

[42] Li H, Y J, Shen Y (2020). Report on the Development of Blood Donors in Shaanxi Province (1998-2018).

[43] Liang J, Li Y, Zhang Z, Shen D, Xu J, Zheng X, Wang T, Tang B, Lei J, Zhang J (2021). Adoption of electronic health records (EHRs) in China during the past 10 years: consecutive survey data analysis and comparison of Sino-American challenges and experiences. J Med Internet Res.

