# Supplementary material for: Exploration of the Healthy Donor Effect Among 0.6 Million Blood Donors in China: Longitudinal Study
Source: JMIR Public Health Surveill. 2024 Feb 22;10:e48617. doi: 10.2196/48617 (PMC10921323; doi:10.2196/48617)
Supplement: Multimedia Appendix 1 [file publichealth_v10i1e48617_app1.docx]

Table S1. Healthy donor effect from the population.

| **ICD-10** | **Participants** | **Non-blood donors pre-donation** | **Incidence**  **(‱)** | **Blood donors pre-donation** (Ref.) | | **Incidence**  **(‱)** | **ARR** | **Lower limit** | **Upper limit** |
| --- | --- | --- | --- | --- | --- | --- | --- | --- | --- |
| **A00-B99** |  |  |  |  |  | |  |  |  |
| 18-25 | 253045 | 582 | 4.49 | 241 | 1.86 | | 2.424 | 2.085 | 2.818 |
| 26-35 | 180185 | 292 | 2.88 | 174 | 1.72 | | 1.665 | 1.374 | 2.016 |
| 36-45 | 164645 | 289 | 3.26 | 184 | 2.08 | | 1.543 | 1.28 | 1.861 |
| 46-55 | 43648 | 112 | 5.54 | 40 | 1.98 | | 3.181 | 2.183 | 4.635 |
| All | 641523 | 1275 | 3.75 | 639 | 1.88 | | 1.995 | 1.812 | 2.196 |
| **C00-D48** |  |  |  |  |  | |  |  |  |
| 18-25 | 253045 | 187 | 1.44 | 122 | 0.94 | | 1.152 | 1.023 | 1.299 |
| 26-35 | 180185 | 249 | 2.46 | 257 | 2.54 | | 0.926 | 0.776 | 1.106 |
| 36-45 | 164645 | 633 | 7.15 | 470 | 5.31 | | 1.381 | 1.091 | 1.617 |
| 46-55 | 43648 | 285 | 14.11 | 113 | 5.59 | | 2.947 | 2.368 | 4.652 |
| All | 641523 | 1354 | 3.99 | 962 | 2.83 | | 1.391 | 1.279 | 1.512 |
| **D50-D89** |  |  |  |  |  | |  |  |  |
| 18-25 | 253045 | 52 | 0.40 | 20 | 0.15 | | 2.737 | 1.618 | 4.631 |
| 26-35 | 180185 | 43 | 0.42 | 15 | 0.15 | | 3.162 | 1.725 | 5.796 |
| 36-45 | 164645 | 70 | 0.79 | 20 | 0.23 | | 3.36 | 2.039 | 5.535 |
| 46-55 | 43648 | 25 | 1.24 | 5 | 0.25 | | 5.181 | 1.968 | 13.636 |
| All | 641523 | 190 | 0.56 | 60 | 0.18 | | 3.225 | 2.402 | 4.33 |
| **E00-E90** |  |  |  |  |  | |  |  |  |
| 18-25 | 253045 | 98 | 0.76 | 61 | 0.47 | | 1.618 | 1.172 | 2.2 |
| 26-35 | 180185 | 191 | 1.89 | 130 | 1.28 | | 1.435 | 1.144 | 1.799 |
| 36-45 | 164645 | 333 | 3.76 | 194 | 2.19 | | 1.762 | 1.471 | 2.109 |
| 46-55 | 43648 | 235 | 11.63 | 82 | 4.06 | | 3.384 | 2.612 | 4.383 |
| All | 641523 | 857 | 2.52 | 467 | 1.37 | | 1.868 | 1.666 | 2.094 |
| **F00-F99** |  |  |  |  |  | |  |  |  |
| 18-25 | 253045 | 136 | 1.05 | 107 | 0.83 | | 1.316 | 1.019 | 1.699 |
| 26-35 | 180185 | 125 | 1.23 | 80 | 0.79 | | 1.549 | 1.165 | 2.059 |
| 36-45 | 164645 | 129 | 1.46 | 78 | 0.88 | | 1.626 | 1.225 | 2.159 |
| 46-55 | 43648 | 37 | 1.83 | 17 | 0.84 | | 2.173 | 1.216 | 3.883 |
| All | 641523 | 427 | 1.26 | 282 | 0.83 | | 1.533 | 1.318 | 1.784 |
| **G00-G99** |  |  |  |  |  | |  |  |  |
| 18-25 | 253045 | 128 | 0.99 | 121 | 0.93 | | 1.057 | 0.823 | 1.358 |
| 26-35 | 180185 | 127 | 1.25 | 170 | 1.68 | | 0.728 | 0.576 | 0.919 |
| 36-45 | 164645 | 248 | 2.80 | 275 | 3.11 | | 0.881 | 0.74 | 1.048 |
| 46-55 | 43648 | 127 | 6.29 | 97 | 4.80 | | 1.319 | 1.007 | 1.729 |
| All | 641523 | 630 | 1.85 | 663 | 1.95 | | 0.939 | 0.841 | 1.049 |
| **I00-I99** |  |  |  |  |  | |  |  |  |
| 18-25 | 253045 | 351 | 2.71 | 283 | 2.18 | | 1.237 | 1.057 | 1.448 |
| 26-35 | 180185 | 563 | 5.56 | 550 | 5.43 | | 0.991 | 0.879 | 1.117 |
| 36-45 | 164645 | 1232 | 13.92 | 1411 | 15.94 | | 0.866 | 0.802 | 0.936 |
| 46-55 | 43648 | 896 | 44.36 | 612 | 30.30 | | 1.493 | 1.343 | 1.66 |
| All | 641523 | 3042 | 8.95 | 2856 | 8.41 | | 1.068 | 1.014 | 1.125 |
| **J00-J99** |  |  |  |  |  | |  |  |  |
| 18-25 | 253045 | 763 | 5.88 | 787 | 6.07 | | 0.974 | 0.881 | 1.077 |
| 26-35 | 180185 | 493 | 4.87 | 613 | 6.05 | | 0.781 | 0.699 | 0.873 |
| 36-45 | 164645 | 625 | 7.06 | 762 | 8.61 | | 0.805 | 0.738 | 0.901 |
| 46-55 | 43648 | 360 | 17.82 | 232 | 11.49 | | 1.55 | 1.31 | 1.835 |
| All | 641523 | 2241 | 6.60 | 2394 | 7.05 | | 0.933 | 0.882 | 1.135 |
| **K00-K93** |  |  |  |  |  | |  |  |  |
| 18-25 | 253045 | 796 | 6.14 | 722 | 5.57 | | 1.102 | 0.995 | 1.219 |
| 26-35 | 180185 | 882 | 8.71 | 965 | 9.53 | | 0.907 | 0.827 | 0.995 |
| 36-45 | 164645 | 1248 | 14.10 | 1207 | 13.63 | | 1.025 | 0.945 | 1.11 |
| 46-55 | 43648 | 536 | 26.54 | 346 | 17.13 | | 1.578 | 1.373 | 1.814 |
| All | 641523 | 3462 | 10.19 | 3240 | 9.54 | | 1.069 | 1.019 | 1.122 |
| **L00-L99** |  |  |  |  |  | |  |  |  |
| 18-25 | 253045 | 120 | 0.93 | 112 | 0.86 | | 1.074 | 0.83 | 1.39 |
| 26-35 | 180185 | 89 | 0.88 | 93 | 0.92 | | 0.906 | 0.675 | 1.218 |
| 36-45 | 164645 | 101 | 1.14 | 101 | 1.14 | | 1.014 | 0.766 | 1.342 |
| 46-55 | 43648 | 48 | 2.38 | 17 | 0.84 | | 2.713 | 1.547 | 4.757 |
| All | 641523 | 358 | 1.05 | 323 | 0.95 | | 1.101 | 0.946 | 1.281 |
| **N00-N99** |  |  |  |  |  | |  |  |  |
| 18-25 | 253045 | 663 | 5.11 | 607 | 4.68 | | 1.089 | 0.975 | 1.216 |
| 26-35 | 180185 | 724 | 7.15 | 836 | 8.25 | | 0.87 | 0.787 | 0.963 |
| 36-45 | 164645 | 837 | 9.45 | 905 | 10.22 | | 0.919 | 0.835 | 1.011 |
| 46-55 | 43648 | 309 | 15.30 | 171 | 8.47 | | 1.856 | 1.532 | 2.248 |
| All | 641523 | 2533 | 7.46 | 2519 | 7.42 | | 1.006 | 0.952 | 1.064 |
| **V01-Y98** |  |  |  |  |  | |  |  |  |
| 18-25 | 253045 | 1 | 0.01 | 0 | 0.00 | | - | - | - |
| 26-35 | 180185 | 6 | 0.06 | 0 | 0.00 | | - | - | - |
| 36-45 | 164645 | 15 | 0.17 | 0 | 0.00 | | - | - | - |
| 46-55 | 43648 | 3 | 0.15 | 0 | 0.00 | | - | - | - |
| All | 641523 | 25 | 0.07 | 0 | 0.00 | | - | - | - |
| **Z00-Z99** |  |  |  |  |  | |  |  |  |
| 18-25 | 253045 | 624 | 4.81 | 278 | 2.14 | | 2.219 | 1.926 | 2.558 |
| 26-35 | 180185 | 363 | 3.58 | 182 | 1.80 | | 1.963 | 1.64 | 2.349 |
| 36-45 | 164645 | 159 | 1.80 | 125 | 1.41 | | 1.269 | 1.003 | 1.607 |
| 46-55 | 43648 | 69 | 3.42 | 25 | 1.24 | | 2.683 | 1.688 | 4.264 |
| All | 641523 | 1215 | 3.58 | 610 | 1.80 | | 1.98 | 1.796 | 2.184 |
| **Ovarall** |  |  |  |  |  | |  |  |  |
| 18-25 | 253045 | 4490 | 34.63 | 3461 | 26.69 | | 1.302 | 1.245 | 1.362 |
| 26-35 | 180185 | 4081 | 40.29 | 4115 | 40.62 | | 0.979 | 0.936 | 1.023 |
| 36-45 | 164645 | 5765 | 65.12 | 5782 | 65.31 | | 0.988 | 0.951 | 1.025 |
| 46-55 | 43648 | 3020 | 149.51 | 1757 | 86.98 | | 1.816 | 1.707 | 1.932 |
| All | 641523 | 17356 | 51.09 | 15115 | 44.50 | | 1.152 | 1.127 | 1.178 |

Table S2. Healthy donor effect by gender differences (male).

| **ICD-10** | **Participants** | **Non-blood donors pre-donation** | **Incidence**  **(‱)** | **Blood donors pre-donation (Ref.)** | **Incidence**  **(‱)** | **ARR** | **Lower limit** | **Upper limit** |
| --- | --- | --- | --- | --- | --- | --- | --- | --- |
| **A00-B99** |  |  |  |  |  |  |  |  |
| 18-25 | 158631 | 423 | 5.18 | 180 | 2.21 | 2.333 | 1.959 | 2.779 |
| 26-35 | 115020 | 210 | 3.47 | 128 | 2.12 | 1.582 | 1.263 | 1.983 |
| 36-45 | 98318 | 194 | 3.98 | 132 | 2.71 | 1.35 | 1.076 | 1.694 |
| 46-55 | 24712 | 71 | 6.92 | 22 | 2.15 | 3.736 | 2.228 | 6.265 |
| All | 396681 | 898 | 4.47 | 462 | 2.30 | 1.89 | 1.686 | 2.118 |
| **C00-D48** |  |  |  |  |  |  |  |  |
| 18-25 | 158631 | 52 | 0.64 | 48 | 0.59 | 1.079 | 0.729 | 1.598 |
| 26-35 | 115020 | 54 | 0.89 | 79 | 1.31 | 0.868 | 0.67 | 1.152 |
| 36-45 | 98318 | 111 | 2.28 | 78 | 1.60 | 1.417 | 1.054 | 1.906 |
| 46-55 | 24712 | 105 | 10.24 | 15 | 1.46 | 7.094 | 4.103 | 12.262 |
| All | 396681 | 322 | 1.60 | 220 | 1.09 | 1.459 | 1.227 | 1.736 |
| **D50-D89** |  |  |  |  |  |  |  |  |
| 18-25 | 158631 | 18 | 0.22 | 10 | 0.12 | 2.007 | 0.902 | 4.469 |
| 26-35 | 115020 | 15 | 0.25 | 9 | 0.15 | 1.829 | 0.766 | 4.368 |
| 36-45 | 98318 | 17 | 0.35 | 9 | 0.18 | 1.762 | 0.776 | 4 |
| 46-55 | 24712 | 9 | 0.88 | 2 | 0.20 | 4.736 | 1.011 | 22.177 |
| All | 396681 | 59 | 0.29 | 30 | 0.15 | 2.042 | 1.299 | 3.21 |
| **E00-E90** |  |  |  |  |  |  |  |  |
| 18-25 | 158631 | 58 | 0.71 | 38 | 0.47 | 1.515 | 1.006 | 2.281 |
| 26-35 | 115020 | 132 | 2.18 | 101 | 1.67 | 1.289 | 0.991 | 1.675 |
| 36-45 | 98318 | 202 | 4.15 | 117 | 2.40 | 1.816 | 1.438 | 2.293 |
| 46-55 | 24712 | 150 | 14.63 | 50 | 4.88 | 3.832 | 2.744 | 5.351 |
| All | 396681 | 542 | 2.70 | 306 | 1.52 | 1.827 | 1.584 | 2.106 |
| **F00-F99** |  |  |  |  |  |  |  |  |
| 18-25 | 158631 | 88 | 1.08 | 73 | 0.89 | 1.247 | 0.911 | 1.707 |
| 26-35 | 115020 | 64 | 1.06 | 51 | 0.84 | 1.222 | 0.84 | 1.778 |
| 36-45 | 98318 | 50 | 1.03 | 34 | 0.70 | 1.428 | 0.922 | 2.212 |
| 46-55 | 24712 | 18 | 1.76 | 5 | 0.49 | 3.513 | 1.288 | 9.577 |
| All | 396681 | 220 | 1.09 | 163 | 0.81 | 1.363 | 1.11 | 1.672 |
| **G00-G99** |  |  |  |  |  |  |  |  |
| 18-25 | 158631 | 100 | 1.23 | 98 | 1.20 | 1.007 | 0.76 | 1.334 |
| 26-35 | 115020 | 83 | 1.37 | 117 | 1.93 | 0.704 | 0.529 | 0.936 |
| 36-45 | 98318 | 148 | 3.04 | 163 | 3.35 | 0.894 | 0.714 | 1.12 |
| 46-55 | 24712 | 67 | 6.53 | 54 | 5.27 | 1.259 | 0.87 | 1.822 |
| All | 396681 | 398 | 1.98 | 432 | 2.15 | 0.916 | 0.798 | 1.051 |
| **I00-I99** |  |  |  |  |  |  |  |  |
| 18-25 | 158631 | 221 | 2.71 | 206 | 2.52 | 1.081 | 0.893 | 1.308 |
| 26-35 | 115020 | 371 | 6.14 | 365 | 6.04 | 0.984 | 0.849 | 1.14 |
| 36-45 | 98318 | 695 | 14.27 | 905 | 18.58 | 0.758 | 0.685 | 0.838 |
| 46-55 | 24712 | 551 | 53.74 | 333 | 32.48 | 1.744 | 1.515 | 2.008 |
| All | 396681 | 1838 | 9.14 | 1809 | 9.00 | 1.026 | 0.961 | 1.096 |
| **J00-J99** |  |  |  |  |  |  |  |  |
| 18-25 | 158631 | 440 | 5.39 | 486 | 5.96 | 0.913 | 0.802 | 1.039 |
| 26-35 | 115020 | 276 | 4.56 | 334 | 5.52 | 0.819 | 0.714 | 0.942 |
| 36-45 | 98318 | 327 | 6.72 | 433 | 8.89 | 0.776 | 0.687 | 0.88 |
| 46-55 | 24712 | 212 | 20.68 | 125 | 12.19 | 1.749 | 1.394 | 2.195 |
| All | 396681 | 1255 | 6.24 | 1378 | 6.85 | 0.916 | 0.854 | 1.024 |
| **K00-K93** |  |  |  |  |  |  |  |  |
| 18-25 | 158631 | 532 | 6.52 | 483 | 5.92 | 1.112 | 0.982 | 1.2 |
| 26-35 | 115020 | 570 | 9.43 | 613 | 10.14 | 0.927 | 0.826 | 1.041 |
| 36-45 | 98318 | 711 | 14.60 | 721 | 14.81 | 0.975 | 0.877 | 1.083 |
| 46-55 | 24712 | 327 | 31.89 | 179 | 17.46 | 1.969 | 1.63 | 2.379 |
| All | 396681 | 2140 | 10.65 | 1996 | 9.93 | 1.081 | 1.016 | 1.15 |
| **L00-L99** |  |  |  |  |  |  |  |  |
| 18-25 | 158631 | 74 | 0.91 | 87 | 1.07 | 0.849 | 0.623 | 1.157 |
| 26-35 | 115020 | 49 | 0.81 | 69 | 1.14 | 0.696 | 0.481 | 1.007 |
| 36-45 | 98318 | 49 | 1.01 | 61 | 1.25 | 0.805 | 0.547 | 1.183 |
| 46-55 | 24712 | 35 | 3.41 | 8 | 0.78 | 4.3 | 1.976 | 9.357 |
| All | 396681 | 207 | 1.03 | 225 | 1.12 | 0.916 | 0.757 | 1.108 |
| **N00-N99** |  |  |  |  |  |  |  |  |
| 18-25 | 158631 | 305 | 3.74 | 305 | 3.74 | 1 | 0.853 | 1.173 |
| 26-35 | 115020 | 239 | 3.95 | 309 | 5.11 | 0.798 | 0.673 | 0.947 |
| 36-45 | 98318 | 256 | 5.26 | 272 | 5.59 | 0.913 | 0.767 | 1.086 |
| 46-55 | 24712 | 129 | 12.58 | 54 | 5.27 | 2.588 | 1.855 | 3.61 |
| All | 396681 | 929 | 4.62 | 940 | 4.68 | 0.99 | 0.903 | 1.084 |
| **V01-Y98** |  |  |  |  |  |  |  |  |
| 18-25 | 158631 | 0 | 0.00 | 0 | 0.00 | - | - | - |
| 26-35 | 115020 | 1 | 0.02 | 0 | 0.00 | - | - | - |
| 36-45 | 98318 | 2 | 0.04 | 0 | 0.00 | - | - | - |
| 46-55 | 24712 | 1 | 0.10 | 0 | 0.00 | - | - | - |
| All | 396681 | 4 | 0.02 | 0 | 0.00 | - | - | - |
| **Z00-Z99** |  |  |  |  |  |  |  |  |
| 18-25 | 158631 | 89 | 1.09 | 135 | 1.65 | 0.657 | 0.502 | 0.86 |
| 26-35 | 115020 | 60 | 0.99 | 99 | 1.64 | 0.601 | 0.434 | 0.832 |
| 36-45 | 98318 | 69 | 1.42 | 77 | 1.58 | 0.894 | 0.645 | 1.24 |
| 46-55 | 24712 | 33 | 3.22 | 14 | 1.37 | 2.273 | 1.205 | 4.289 |
| All | 396681 | 251 | 1.25 | 325 | 1.62 | 0.765 | 0.649 | 0.903 |
| **Ovarall** |  |  |  |  |  |  |  |  |
| 18-25 | 158631 | 2399 | 29.40 | 2149 | 26.33 | 1.124 | 1.059 | 1.192 |
| 26-35 | 115020 | 2108 | 34.86 | 2324 | 38.43 | 0.956 | 0.919 | 1.015 |
| 36-45 | 98318 | 2797 | 57.44 | 3052 | 62.67 | 0.963 | 0.937 | 1.034 |
| 46-55 | 24712 | 1706 | 166.39 | 861 | 83.97 | 2.193 | 2.011 | 2.392 |
| All | 396681 | 9010 | 44.82 | 8386 | 41.72 | 1.082 | 1.05 | 1.116 |

Table S3. Healthy donor effect by gender differences (female).

| **ICD-10** | **Participants** | **Non-blood donors pre-donation** | **Incidence**  **(‱)** | **Blood donors pre-donation (Ref.)** | **Incidence**  **(‱)** | **ARR** | **Lower limit** | **Upper limit** |
| --- | --- | --- | --- | --- | --- | --- | --- | --- |
| **A00-B99** |  |  |  |  |  |  |  |  |
| 18-25 | 94414 | 159 | 3.31 | 61 | 1.27 | 2.647 | 1.965 | 3.565 |
| 26-35 | 65165 | 82 | 2.01 | 46 | 1.13 | 1.81 | 1.252 | 2.617 |
| 36-45 | 66327 | 95 | 2.38 | 52 | 1.31 | 1.754 | 1.243 | 2.476 |
| 46-55 | 18936 | 41 | 4.12 | 18 | 1.81 | 2.497 | 1.427 | 4.369 |
| All | 244842 | 377 | 2.72 | 177 | 1.28 | 2.134 | 1.781 | 2.557 |
| **C00-D48** |  |  |  |  |  |  |  |  |
| 18-25 | 94414 | 135 | 2.81 | 74 | 1.54 | 1.793 | 1.345 | 2.391 |
| 26-35 | 65165 | 195 | 4.78 | 178 | 4.36 | 1.038 | 0.844 | 1.277 |
| 36-45 | 66327 | 522 | 13.10 | 392 | 9.84 | 1.308 | 1.145 | 1.493 |
| 46-55 | 18936 | 180 | 18.10 | 98 | 9.85 | 1.92 | 1.493 | 2.471 |
| All | 244842 | 1032 | 7.44 | 742 | 5.35 | 1.362 | 1.238 | 1.498 |
| **D50-D89** |  |  |  |  |  |  |  |  |
| 18-25 | 94414 | 34 | 0.71 | 10 | 0.21 | 3.336 | 1.647 | 6.758 |
| 26-35 | 65165 | 28 | 0.69 | 6 | 0.15 | 4.916 | 2.033 | 11.8 |
| 36-45 | 66327 | 53 | 1.33 | 11 | 0.28 | 4.683 | 2.443 | 8.978 |
| 46-55 | 18936 | 16 | 1.61 | 3 | 0.30 | 5.447 | 1.577 | 18.809 |
| All | 244842 | 131 | 0.94 | 30 | 0.22 | 4.294 | 2.887 | 6.388 |
| **E00-E90** |  |  |  |  |  |  |  |  |
| 18-25 | 94414 | 40 | 0.83 | 23 | 0.48 | 1.767 | 1.047 | 2.983 |
| 26-35 | 65165 | 59 | 1.45 | 29 | 0.71 | 1.969 | 1.252 | 3.097 |
| 36-45 | 66327 | 131 | 3.29 | 77 | 1.93 | 1.703 | 1.283 | 2.261 |
| 46-55 | 18936 | 85 | 8.55 | 32 | 3.22 | 2.823 | 1.871 | 4.259 |
| All | 244842 | 315 | 2.27 | 161 | 1.16 | 1.954 | 1.613 | 2.367 |
| **F00-F99** |  |  |  |  |  |  |  |  |
| 18-25 | 94414 | 48 | 1.00 | 34 | 0.71 | 1.417 | 0.913 | 2.201 |
| 26-35 | 65165 | 61 | 1.49 | 29 | 0.71 | 2.121 | 1.36 | 3.309 |
| 36-45 | 66327 | 79 | 1.98 | 44 | 1.10 | 1.837 | 1.265 | 2.668 |
| 46-55 | 18936 | 19 | 1.91 | 12 | 1.21 | 1.575 | 0.76 | 3.267 |
| All | 244842 | 207 | 1.49 | 119 | 0.86 | 1.755 | 1.399 | 2.202 |
| **G00-G99** |  |  |  |  |  |  |  |  |
| 18-25 | 94414 | 28 | 0.58 | 23 | 0.48 | 1.255 | 0.717 | 2.195 |
| 26-35 | 65165 | 44 | 1.08 | 53 | 1.30 | 0.782 | 0.521 | 1.175 |
| 36-45 | 66327 | 100 | 2.51 | 112 | 2.81 | 0.86 | 0.654 | 1.13 |
| 46-55 | 18936 | 60 | 6.03 | 43 | 4.32 | 1.406 | 0.945 | 2.093 |
| All | 244842 | 232 | 1.67 | 231 | 1.67 | 0.98 | 0.816 | 1.178 |
| **I00-I99** |  |  |  |  |  |  |  |  |
| 18-25 | 94414 | 130 | 2.70 | 77 | 1.60 | 1.627 | 1.226 | 2.1 |
| 26-35 | 65165 | 192 | 4.70 | 185 | 4.53 | 1.01 | 0.823 | 1.24 |
| 36-45 | 66327 | 537 | 13.48 | 506 | 12.70 | 1.061 | 0.938 | 1.199 |
| 46-55 | 18936 | 345 | 34.69 | 279 | 28.05 | 1.221 | 1.039 | 1.434 |
| All | 244842 | 1204 | 8.68 | 1047 | 7.55 | 1.14 | 1.049 | 1.239 |
| **J00-J99** |  |  |  |  |  |  |  |  |
| 18-25 | 94414 | 323 | 6.72 | 301 | 6.26 | 1.056 | 0.901 | 1.236 |
| 26-35 | 65165 | 217 | 5.31 | 279 | 6.83 | 0.752 | 0.628 | 0.9 |
| 36-45 | 66327 | 298 | 7.48 | 329 | 8.26 | 0.899 | 0.768 | 1.054 |
| 46-55 | 18936 | 148 | 14.88 | 107 | 10.76 | 1.343 | 1.043 | 1.729 |
| All | 244842 | 986 | 7.11 | 1016 | 7.33 | 0.961 | 0.88 | 1.05 |
| **K00-K93** |  |  |  |  |  |  |  |  |
| 18-25 | 94414 | 264 | 5.49 | 239 | 4.97 | 1.08 | 0.905 | 1.288 |
| 26-35 | 65165 | 312 | 7.64 | 352 | 8.62 | 0.879 | 0.753 | 1.025 |
| 36-45 | 66327 | 537 | 13.48 | 486 | 12.20 | 1.1 | 0.972 | 1.245 |
| 46-55 | 18936 | 209 | 21.01 | 167 | 16.79 | 1.217 | 0.989 | 1.498 |
| All | 244842 | 1322 | 9.53 | 1244 | 8.97 | 1.053 | 0.974 | 1.138 |
| **L00-L99** |  |  |  |  |  |  |  |  |
| 18-25 | 94414 | 46 | 0.96 | 25 | 0.52 | 1.881 | 1.155 | 3.064 |
| 26-35 | 65165 | 40 | 0.98 | 24 | 0.59 | 1.479 | 0.882 | 2.478 |
| 36-45 | 66327 | 52 | 1.31 | 40 | 1.00 | 1.339 | 0.884 | 2.03 |
| 46-55 | 18936 | 13 | 1.31 | 9 | 0.90 | 1.319 | 0.552 | 3.153 |
| All | 244842 | 151 | 1.09 | 98 | 0.71 | 1.519 | 1.175 | 1.963 |
| **N00-N99** |  |  |  |  |  |  |  |  |
| 18-25 | 94414 | 358 | 7.45 | 302 | 6.28 | 1.155 | 0.99 | 1.347 |
| 26-35 | 65165 | 485 | 11.88 | 527 | 12.91 | 0.911 | 0.804 | 1.033 |
| 36-45 | 66327 | 581 | 14.58 | 633 | 15.89 | 0.922 | 0.823 | 1.033 |
| 46-55 | 18936 | 180 | 18.10 | 117 | 11.76 | 1.549 | 1.223 | 1.963 |
| All | 244842 | 1604 | 11.57 | 1579 | 11.39 | 1.011 | 0.939 | 1.08 |
| **V01-Y98** |  |  |  |  |  |  |  |  |
| 18-25 | 94414 | 1 | 0.02 | 0 | 0.00 | - | - | - |
| 26-35 | 65165 | 5 | 0.12 | 0 | 0.00 | - | - | - |
| 36-45 | 66327 | 13 | 0.33 | 0 | 0.00 | - | - | - |
| 46-55 | 18936 | 2 | 0.20 | 0 | 0.00 | - | - | - |
| All | 244842 | 21 | 0.15 | 0 | 0.00 | - | - | - |
| **Z00-Z99** |  |  |  |  |  |  |  |  |
| 18-25 | 94414 | 535 | 11.13 | 143 | 2.98 | 3.58 | 2.975 | 4.308 |
| 26-35 | 65165 | 303 | 7.42 | 83 | 2.03 | 3.594 | 2.812 | 4.594 |
| 36-45 | 66327 | 90 | 2.26 | 48 | 1.20 | 1.867 | 1.314 | 2.653 |
| 46-55 | 18936 | 36 | 3.62 | 11 | 1.11 | 3.178 | 1.607 | 6.285 |
| All | 244842 | 964 | 6.95 | 285 | 2.06 | 3.354 | 2.937 | 3.829 |
| **Ovarall** |  |  |  |  |  |  |  |  |
| 18-25 | 94414 | 2091 | 43.51 | 1312 | 27.30 | 1.571 | 1.465 | 1.685 |
| 26-35 | 65165 | 1973 | 48.32 | 1791 | 43.86 | 1.085 | 1.017 | 1.159 |
| 36-45 | 66327 | 2968 | 74.50 | 2730 | 68.53 | 1.083 | 1.027 | 1.143 |
| 46-55 | 18936 | 1314 | 132.11 | 896 | 90.09 | 1.492 | 1.365 | 1.63 |
| All | 244842 | 8346 | 60.18 | 6729 | 48.52 | 1.236 | 1.196 | 1.277 |
